# Oral Complaint Visits to the Pediatric Emergency Department During the COVID-19 Pandemic

**DOI:** 10.7759/cureus.28559

**Published:** 2022-08-29

**Authors:** Jenelle Fleagle, Wendi Xiao, Michael Cottam, Margarita Lorch

**Affiliations:** 1 Department of Dentistry, Nemours Children's Health System, Wilmington, USA; 2 Department of Biomedical Research, Nemours Children's Health System, Wilmington, USA; 3 Division of Emergency Medicine, Nemours Children's Health System, Wilmington, USA

**Keywords:** sars-cov-2, pediatric, emergency department, dentistry, covid-19

## Abstract

Objective: We aimed to describe differences in orofacial complaints presenting to a pediatric emergency department (PED) during the COVID-19 pandemic as compared to those presenting prior to the pandemic.

Study design: A retrospective review was conducted in the PED from March 16, 2020, to August 16, 2020, and compared with the prior year.

Results: Despite a 41% reduction in total PED visits, oral visits as a percentage of PED volume increased (3% vs 2%) P < 0.01) during the pandemic. More children with dental complaints required intervention during the pandemic (48% vs 30%, P < 0.001) including extractions and splinting (15% vs 1%, P < 0.001). Compared with pre-pandemic, proportion of tooth infections increased (68% vs 40%, P < 0.001), while oral ulcers decreased (19% vs 47%, P < 0.001).

Conclusion: Pediatric emergency department presentation decreased during the pandemic, but patients requiring interventions increased. This may reflect hesitation in seeking treatment, outpatient facility closures, and increased acuity at the time of PED presentation due to delays in seeking care.

## Introduction

The United States declared a national emergency on March 13, 2020, because of the coronavirus disease 2019 (COVID-19) [1]. This led to national closures of all nonemergency medical services including dental services. The emergency department (ED) became the only treatment option for many patients.

Many dental offices remained closed during the initial COVID-19 pandemic with limited hours throughout the spring of 2020 [2]. The American Dental Association (ADA) Health Policy Institute completed a biweekly poll the week of March 23 which showed 76% of dentists surveyed had closed their offices to all but emergency patients, and another 19% indicated their offices were closed completely [3]. Prior to the pandemic, many dental emergencies such as splinting and extractions, which require timely intervention, were triaged in the ED to dental offices. The pandemic caused a unique situation in that the ED was limited in all referral resources, thus increasing the need for treatment to be provided within the ED [4]. Historical studies have noted the difficulty of providing dental services within the ED, with many triaging and directing treatment to dental clinics [5,6]. The purpose of this study is to describe differences in pediatric oral complaints presenting to a pediatric emergency department (PED) during the COVID-19 pandemic as compared with the pre-pandemic era in presentation, treatment, and outcomes.

## Materials and methods

A retrospective cohort study was performed at Alfred I. duPont Hospital for Children in Wilmington, DE, from March 16 to August 16, 2020, in a comparison with the same period in 2019 after obtaining approval from the Nemours Institutional Review Board (approval number 1646577).

The collection of ED visits was analyzed using a subset of records with a primary and secondary billable diagnosis (International Classification of Disease, Tenth Revision {ICD-10}). All patients from 0 to 19 years of age with an oral complaint who visited our hospital’s ED were evaluated for inclusion in this study. Patients with multisystem trauma, currently prescribed amoxicillin for otitis media with oral ulcerations, and nonodontogenic swellings were excluded. Multisystem trauma was defined as involving more than one system located outside of the direct oral vicinity. In the event a patient presented multiple times to the PED for an oral complaint during the study period, only the initial visit was recorded.

Potential patients were initially identified using QlikView (Qlik, King of Prussia, PA). These patient medical records were then reviewed for eligibility, extracted, and entered by one researcher (JF). The records were transferred to data collection sheets removing all personal information. A pilot sample was initially conducted to test for data collection accuracy by comparing Qlikview results with resident logbooks and EPIC workbench (EPIC Systems Corporation, Veroina, WI) for accuracy. Any conflicts between these modalities were reviewed by a second researcher (ML) to assess for eligibility.

Demographic information was summarized using the median and interquartile ranges for continuous variables and frequency, and percentages for categorical variables. The dental complaints and interventions were summarized using frequency and percentage for each item, and statistical analysis was carried out using Pearson’s chi-square test and t-test when appropriate to detect any difference between 2019 and 2020. All tests were two-tailed, and a P-value less than 0.05 was regarded as significant. The statistical software used for analysis was R, version 3.6.2 (R is an open-source software program by R Development Core Team).

## Results

**Figure 1:**
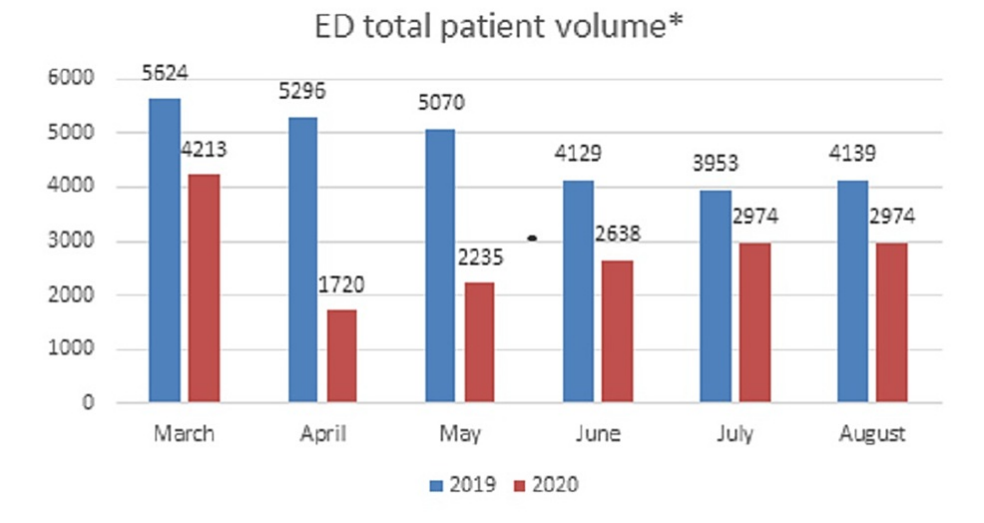
ED total patient volume Total pediatric emergency department (ED) patient volume.

**Figure 2:**
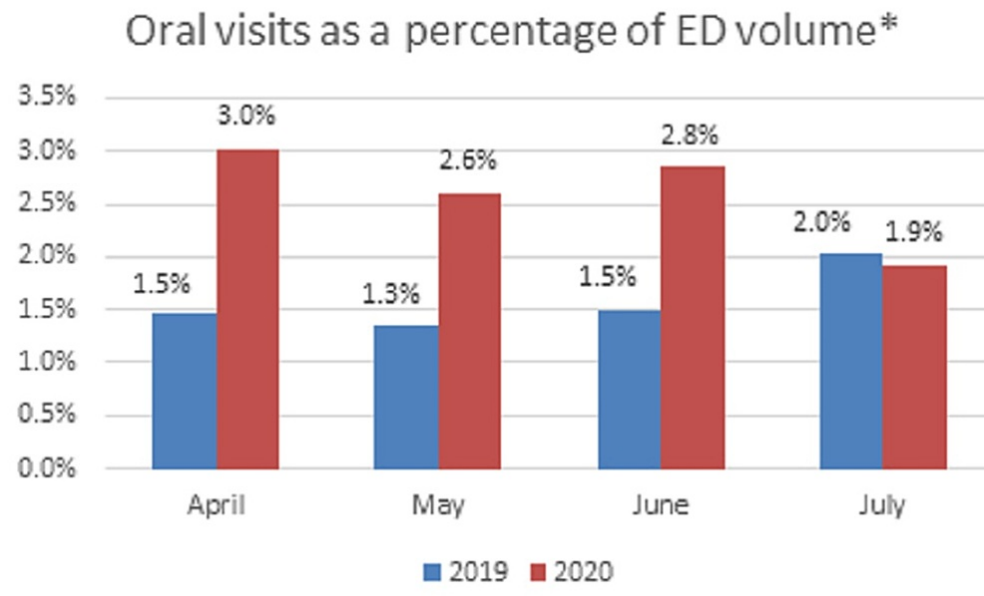
Oral visits as a percentage of ED volume Visits associated with an oral ICD-10 diagnosis in relation to total patient volume. ED = emergency department; ICD-10 = International Classification of Disease, Tenth Revision

We identified 937 patients with oral complaints during the study periods. Of these, 680 patients met inclusion criteria. Patient numbers decreased from 374 in 2019 to 306 in 2020, which follows the overall ED visit trend encountered during the pandemic [1,7]. The ED patient volume significantly differed between the study periods with an average 41% reduction (P < 0.01) in total ED visits (Figure 1). The month of April was the lowest patient volume presenting to the ED with the highest presentation of oral chief complaints by percentage (Figure 2). Patients presenting with an oral complaint accounted for an average of 2% in 2019 and 3% in 2020 of total ED visits with 4.4% (n = 30/680) requiring admission during the study period.

In 2019, the admission rate for oral complaints was 5.1% (n = 19). Diagnoses requiring admission included postoperative bleeding, dental infection, stomatitis/oral ulcerations, and oral trauma. In 2020, the admission rate for oral complaints was 3.6% (n = 11) and included postoperative bleeding, dental infections, stomatitis, and oral trauma patients. No statistically significant difference in admissions was found between the periods (P = 0.95) or for patients presenting with swelling and dental pain (2019 77.3% {n = 289} to 2020 74.8% {n = 229}).

Demographically, there was no statistically significant difference between the study periods (Table 1). The mean age for both periods was five years. Males accounted for the majority of patients in both study periods (61.2% and 57.8%). Visits to the PED for oral complaints during the pandemic increased among those identifying as Caucasian but decreased for all other races and ethnicities. No further analysis on racial differences was completed because of missing demographic information. Medicaid (the United States social health care program) made up the majority of patient insurance at 51.9% (n = 353/680). Although not statistically significant, private insurance increased (41.4% vs 46.4%), while Medicaid (53.5% vs 50% ) and self-pay (5.1% vs 3.6% ) showed a decreasing trend.

**Table 1:** Patient demographics *Denotes statistical significance.

Demographics		2019 (n = 374)	2020 (n = 306)
Age			
	Median	5.00	4.00
	(Interquartile range)	(2.00-8.00)	(2.00-8.00)
Insurance			
	Medicaid	200 (53.5%)	153 (50.0%)
	Private	155 (41.4%)	142 (46.4%)
	Self-pay	19 (5.1%)	11 (3.6%)
Sex			
	Female	145 (38.8%)	129 (42.2%)
	Male	229 (61.2%)	177 (57.8%)
Race*			
	Black	112 (29.9%)	78 (25.5%)
	White	188 (50.3%)	180 (58.8%)
	Hispanic	47 (12.6%)	23 (7.5%)
	Other	17 (4.5%)	17 (5.6%)
	Unknown	10 (2.7%)	8 (2.6%)

Oral trauma comprised 67.4% and 68% of visits in 2019 and 2020, respectively (Table 2). Oral infections made up 28.1% (n = 105) of oral complaints in 2019 and 23.5% (n = 72) in 2020. Overall, differences in oral trauma and infection presentations were not found to be statistically significant (P = 0.95) between the study periods.

**Table 2:** Etiology and interventions of dental complaints treated in the pediatric emergency department. *Denotes statistical significance.

Dental Complaint	2019	2020
Swelling/Pain	85 (22.7%)	77 (25.2%)
Infection	105 (28.1%)	72 (23.5%)
Oral ulcers*	50 (47.6%)	14 (19.4%)
Tooth abscess*	42 (40.0%)	49 (68.1%)
Trauma	252 (67.4%)	208 (68.0%)
Bike accident*	9 (3.6%)	20 (9.6%)
Dog bite	15 (6%)	18 (8.7%)
Sports/school*	55 (21.8%)	4 (1.9%)
Intervention*	2019	2020
Any intervention	113 (30.2%)	147 (48.0%)
Extraction*	4 (1.1%)	45 (14.7%)
Antibiotics	49 (13.1%)	47 (15.4%)
Sutures	72 (19.3%)	69 (22.5%)
Splint/composite*	1 (0.3%)	11 (3.6%)
Admission	19 (5.1%)	11 (3.6%)

Total oral infections were further separated into oral ulcerations, which consisted of stomatitis or associated teething/eruption diagnosis, and tooth infections. Oral ulcerations and tooth infections showed a significant difference in presentation rates (P < 0.001) between the study periods. Oral ulcerations comprised 47.6% (n = 50) of oral infection visits in 2019 and 19.4% (n = 14) in 2020, and tooth infections comprised 40% (n = 42) of oral infection visits in 2019 and 68.1% (n = 49) in 2020.

Oral trauma was further separated into frequently encountered categories. Dog bites accounted for 6% (n = 15) of oral trauma in 2019 and 8.7% (n = 18) in 2020, which was not found to be statistically significant (P = 0.95). Bicycle accidents comprised 3.6% (n = 9) of oral trauma visits in 2019 and 9.6% (n = 20) in 2020 (P = 0.014). Sports/school activities made up 21.8% (n = 55) of oral trauma visits in 2019 and 1.9% (n = 4) in 2020 (P < 0.001).

**Figure 3:**
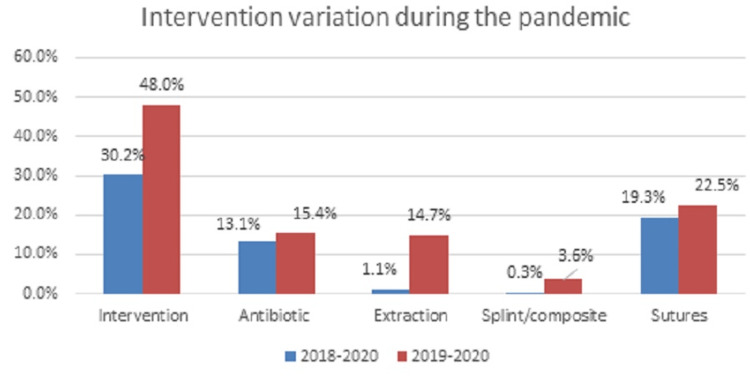
Intervention variation during the pandemic Total intervention provided between the two study years.

Treatment interventions provided in the ED differed significantly between the study periods (P < 0.001) (Table 2). Interventions provided in the PED included tooth extractions, antibiotics, sutures, and splint/composite temporary (Figure 3). Statistical significance was found for only extractions (P < 0.001) and splint/composite temporary (P = 0.003).

The most common ICD-10 principal diagnosis between the study periods remained a laceration of the lip, followed by a laceration of the oral cavity. Laceration repairs requiring sutures accounted for 20.7% (n = 141/680) or one in five of the total patient volumes presenting with an oral complaint.

## Discussion

In the early pandemic, new regulations were placed on medical services because of COVID-19, which likely created additional stress and limitations leading to access to care issues for years to come [1,8]. This study is consistent with previous ED studies during the early four-week interval of the pandemic showing a substantial reduction in ED visits during the same period when compared with the previous year [1,7]. The pandemic resulted in more patients presenting with oral infections and pain being referred by their dental provider because of office closures. All elective general anesthesia cases were canceled until June 1, 2020, further delaying dental treatment for patients requiring sedation. Because of pre-appointment screening questionnaires, oral infections with systemic involvement further complicated patients presenting to their dental offices, likely leading more patients to be referred to the ED for evaluation [9].

During the early months of the pandemic, we saw a greater percentage of patients requiring extractions in our PED. Several patients, who were excluded from our study, also returned for follow-up visits requiring extractions, admissions, or both because of failure of antibiotic therapy. In addition, during the study period, two patients were diagnosed with COVID-19 prior to their inpatient admission [4]. COVID-19 exposure at our hospital was always considered a possibility during aerosol-generating procedures; thus, regardless of symptoms, we retained and followed strict personal protective equipment guidelines. To our knowledge, no team member contracted COVID-19 during the extractions and procedural sedation of these patients.

Interestingly, oral injuries remained the same throughout both periods even without formal scholastic activities. Because of this finding, we wanted to further isolate specific etiologies such as dog bites, school/sports injuries, and bicycle injuries. Historically, bite wounds affecting the face account for about 15% of total bite wounds in the United States [10]. We found dog bite prevalence to account for 5% of total patients presenting with an oral complaint to our PED, keeping in mind our exclusion protocol likely prevented a higher prevalence. Further breakdown of the patients presenting with trauma shows that dog bites accounted for 6% of all traumatic wounds involving the oral cavity in 2019 and 8.75% in 2020. Although the increase was not statistically significant, we speculate the bites increased because of stay-at-home orders leading to more occupants in the home for longer periods of time, likely stressing the pet. The increase in bicycle injuries, which is the most common child consumer sports product related to dental injury [11], and the decrease in sport/school injuries are likely also strongly correlated to the stay-at-home orders and school closures in place at this time. 

During the pandemic, oral treatment interventions increased. We speculate the increase was due to the acuity at presentation and lack of referral sources. Historically, most ED visits for a dental assessment result in comfort-care measures rather than definitive care [12]. During this study, the need for extractions and antibiotics increased, which correlates to previous ED trends [1]. Furthermore, providing definitive treatment in the form of dental extractions at the time of service decreases antibiotic prescription indications; thus, we attribute the increase in antibiotics to acuity at presentation. We also found hospital inpatient admissions during our study period were similar to those of past studies [13]. During the pandemic, there was a decrease in inpatient admission rates between the periods. We account for this trend by providing treatment in the ED (suturing and extractions in the ED vs operating room), patient fear of presenting to the ED, and conservation of medical resources.

Dental trauma is a common type of pediatric trauma presenting to the ED with the highest incidence of dental trauma estimated to occur between the ages of two to four years in primary dentition and eight to 10 years in secondary dentition [14]. In our study, the average age was five years for both periods; however, we included gingival stomatitis as a diagnosis, which is often excluded from many dental ED studies. Gingival stomatitis is commonly diagnosed between the ages of six months to five years [15]. Interestingly, we saw a significant decrease in patients presenting with this diagnosis during the pandemic, which may be related to overall lower infection rates because of stay-at-home orders [16]. There was a significant decrease in pediatric acute respiratory illnesses like respiratory syncytial virus and influenza, suggesting community mitigation measures lowered additional viral infections [17]. Additionally, oral ulceration has been found to occur with a COVID-19 diagnosis [18,19]. It is not known if COVID-19 oral side effects are the same for children and adults; however, with COVID-19 manifestations, we expect the symptoms in most healthy children to be less or asymptomatic [20,21]. We did not evaluate this association because of limitations in the retrospective nature of our study but suspect disruption to oral tissues, like in many systematic diseases, may correlate to a poorer prognosis, which is less likely in children.

Limitations to this study include the retrospective nature of the study, possible inaccuracies in ICD-10 coding or reported chief compliant, and facial swelling that is later diagnosed as dental etiology upon admission. In addition, the COVID-19 pandemic has shown a greater impact on the pediatric population in the form of child abuse and neglect (CAN) within the home [22]. We identified two children in each period with suspected CAN; however, our exclusion criteria likely excluded additional patients. It is estimated that 50% to 75% of CAN victims include oral trauma in the form of a torn labial frenula, bruising of the labial sulcus, and multiple traumatic incidents [23]. In future studies, it may be helpful to further analyze types of dental diagnosis with visit frequency, CAN victims involving the oral cavity, and underlying medical complexity of the patients presenting with an oral complaint in the PED. Lastly, analyzing referral trends would be beneficial in the evaluation of the pre-appointment screening questionnaire, possibly increasing the recommendation for ED assessment.

## Conclusions

The COVID-19 pandemic significantly decreased overall patient presentation to the ED, which is likely reflective of hesitation to seek treatment. Limitations in referrals to outpatient facilities and fear of COVID-19 exposure likely increased interventions in the PED. The total oral trauma presenting to the PED remained the same between the study periods. Regardless of the venue, children still presented with oral trauma. During the early pandemic, a greater percentage of children with dental complaints required intervention, highlighting the impact of closures of dental clinics to preserve personal protective equipment and prevent aerosol-generating procedures. These findings emphasize the importance of having appropriate access to pediatric dental care and intervention.
